# A study of photoluminescence properties and performance improvement of Cd-doped ZnO quantum dots prepared by the sol–gel method

**DOI:** 10.1186/1556-276X-7-405

**Published:** 2012-07-18

**Authors:** Jun Zhang, Su-Qing Zhao, Kun Zhang, Jian-Qing Zhou, Yan-Fei Cai

**Affiliations:** 1 Faculty of Chemical Engineering and Light Industry, Guangdong University of Technology, Guangzhou, 510006, People’s Republic of China; 2 College of Natural Resources and Environment, South China Agricultural University, Guangzhou, 510642, People’s Republic of China

**Keywords:** Cd-doped ZnO, Quantum dots, Sol–gel method, Photoluminescence properties

## Abstract

In the present work, ZnO quantum dots (QDs) have been prepared by the sol–gel method, and the performance of the QDs has been improved. The effect of Cd concentration on the structural and luminescent properties of the QDs, as well as the effect of the mass ratio of trioctylphosphine oxide (TOPO)/octadecylamine (ODA), has been investigated. The ZnO and Cd-doped ZnO QDs have hexagonal wurtzite structures and are 3 to 6 nm in diameter. When the Cd content was increased, the QD particle size was reduced; this effect was confirmed in the corresponding ultraviolet–visible spectra. The fluorescence intensity was simultaneously enhanced significantly. Both the UV and fluorescence spectra were blue-shifted. The luminous intensity was further enhanced when the QDs were modified with TOPO/ODA. Fourier transform infrared and X-ray diffraction techniques proved that the polymer successfully coated the surfaces of the QDs. A TOPO/ODA mass ratio of 1:2 was determined to result in the best optical performance among the different ratios examined. The results showed that the described synthetic method is appropriate for the preparation of doped QDs with high-fluorescence quantum efficiency.

## Background

Sol–gel-derived materials have received particular interest as chemical receptor matrices because of their optical transparency, mechanical stability, chemical resistance and flexibility in sensor morphological configurations. The materials have a wide spectrum of advantages: they may be set up at room temperature, they are not degraded electrochemically or by light, they are open to a wide variety of chemical modifications, they can be obtained in a variety of forms (monoliths, thin films, fibres or powders), and they are able to respond rapidly. The combination of these factors makes these materials desirable for use in the pharmaceutical, food and chemical industries [1].

Semiconductor quantum dots, also known as semiconductor nanocrystals, are semiconductor materials with a particle size similar to that of the exciton Bohr radius or the de Broglie wavelength [2]. These quantum dots (QDs) have attracted considerable attention from researchers because of their unique optical and electronic properties and have become a bright spot in nanotechnology research [3-9].

ZnO is a direct and wide-band-gap semiconductor material of group II-VI with a hexagonal wurtzite structure (*a* = 0.325 nm, *c* = 0.521 nm). As a result, ZnO has unique electrical and optical properties, such as a wide band gap (Eg = 3.37 eV) at room temperature and a large exciton binding energy of 60 meV, which can produce a significant quantum confinement effect [10]. Particles aggregate easily during the preparation of ZnO QDs because of their large specific surface area and high surface activity. This aggregation can create an irregular surface, which causes many disadvantages in the final products. These issues have an intense effect on the luminescence properties of ZnO QDs. A useful approach to solving this problem is to dope ZnO QDs with another metal. CdO belongs to the cubic system (*a* = 0.467 nm) of II-VI direct-band-gap semiconductor materials and exhibits a band gap of 2.3 eV [11]. The formation of Zn_1-*x*_Cd_*x*_O alloys from ZnO and CdO can cause the band gap of ZnO to red-shift into the blue and green spectral range. The suitable incorporation of Cd enables the band gap to be tuned for various potential applications [12]. In contrast, the lattice mismatch between ZnO and a Zn_1-*x*_Cd_*x*_O alloy with a lower Cd content is small, and this feature allows the preparation of Zn_1-*x*_Cd_*x*_O/ZnO heterojunctions [13].

We previously synthesised ZnO QDs capped with Cd using the sol–gel method in a dilute, water-free solution with PVP K30 (Damao Chemical Reagent Co., Tianjin, China) as a stabiliser and thiourea as a surface modification agent. The optimisation of the Cd matrix and the mass ratio of trioctylphosphine oxide (TOPO)/octadecylamine (ODA) on the optical properties of ZnO QDs have not yet been investigated. In this paper, the Cd content in the alloy was intentionally enhanced in an effort to improve the fluorescence efficiency and stability of the resulting QDs. A comparison between modified QDs and unmodified Cd-doped ZnO QDs is discussed below.

## Methods

Zinc acetate dihydrate (99.0%), cadmium acetate dihydrate (99.5%) and lithium hydroxide (90.0%) were procured from Kermel Chemical Reagent Co. (Tianjin, China). Absolute ethanol (99.7%) and *n*-hexane (98.0%), produced by Zhiyuan Chemical Reagent Co. (Tianjin, China), were used to synthesise Cd-doped ZnO QDs. Octadecylamine, 1-octadecene and TOPO, produced by Aladdin Chemistry Co. (Shanghai, China), were used to modify Cd-doped ZnO QDs. All chemicals were directly used without further treatment.

In a typical sol–gel method, appropriate amounts of Zn(CH_3_COO)_2_·2H_2_O and Cd(CH_3_COO)_2_·2H_2_O were placed in 100 mL of anhydrous ethanol and heated to reflux at 80°C for 3 h. The appropriate amount of Cd(CH_3_COO)_2_·2H_2_O was then added in accordance with the desired molar ratio (*x* = Cd / (Cd + Zn) = 0.0%, 2.0%, 5.0%, 10.0%, or 20.0%) to bring the total amounts of Zn(CH_3_COO)_2_·2H_2_O and Cd(CH_3_COO)_2_·2H_2_O to 15 mmol. Simulta-neously, LiOH (0.3024 g) was dissolved in 20 mL of anhydrous ethanol and kept in an ultrasonic bath for 30 min. This solution was slowly added to the Zn^2+^ and Cd^2+^ solution under a reflux condenser at 50°C for 1 h. The solution was repeatedly washed with *n*-hexane (volume ratio = 1:2) to remove unwanted ions, and the obtained precipitate was dried in a vacuum oven to afford Cd-doped ZnO QDs as white powders. Next, 1-octadecene, octadecylamine, TOPO and Cd-doped ZnO QDs were slowly mixed in anhydrous ethanol with constant stirring at 50°C for 1 h. The transparent solution turned into a white solid when cooled to room temperature and was washed repeatedly with chloroform to provide the final product.

The synthesised powder samples were dissolved in ethanol, and a drop of this dilute ethanolic solution was placed on the surface of a copper grid. Microstructural investigations of dried samples were performed using a transmission electron microscope (TEM, JEM-2010, JEOL Ltd., Akishima-shi, Japan) with an acceleration voltage of 200 kV. Optical absorption measurements in the UV-visible range were performed at room temperature using a TU-1901 spectrophotometer equipped with a deuterium lamp as a UV light source and a tungsten halogen lamp as a visible source (Beijing Purkinje General Instrument Co., Ltd., Beijing, China). The wavelength range used in the experiment was 200 to 600 nm. X-ray diffraction (XRD) profiles of QD samples were obtained on an Ultima-III apparatus equipped with Cu Kα radiation source (Rigaku Corporation, Beijing, China). For fluorescence spectroscopy measurements, the excitation wavelength for QDs was identified as 341 nm using a Hitachi F-7000 fluorimeter (Chiyoda-ku, Japan). Samples were prepared using the KBr press disc method, and optical transmittance was determined by Fourier transform infrared (FT-IR) analysis. In addition, a Canon EOS-500D (Ohta-ku, Japan) was used for digital photos under a fluorescent lamp and under UV light at room temperature.

## Results and discussion

**Figure 1:**
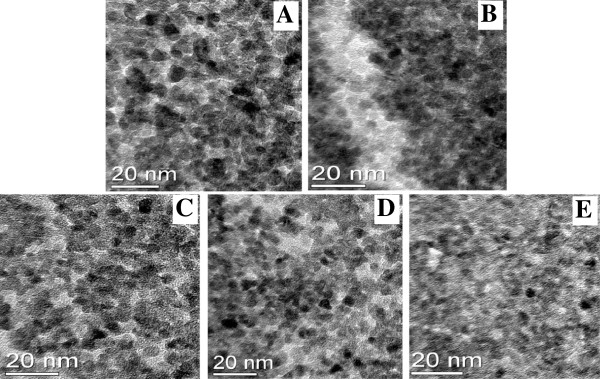
**TEM images.** ZnO QDs doped with (**A**) 0%, (**B**) 2%, (**C**) 5%, (**D**) 10% and (**E**) 20% Cd.

**Figure 2:**
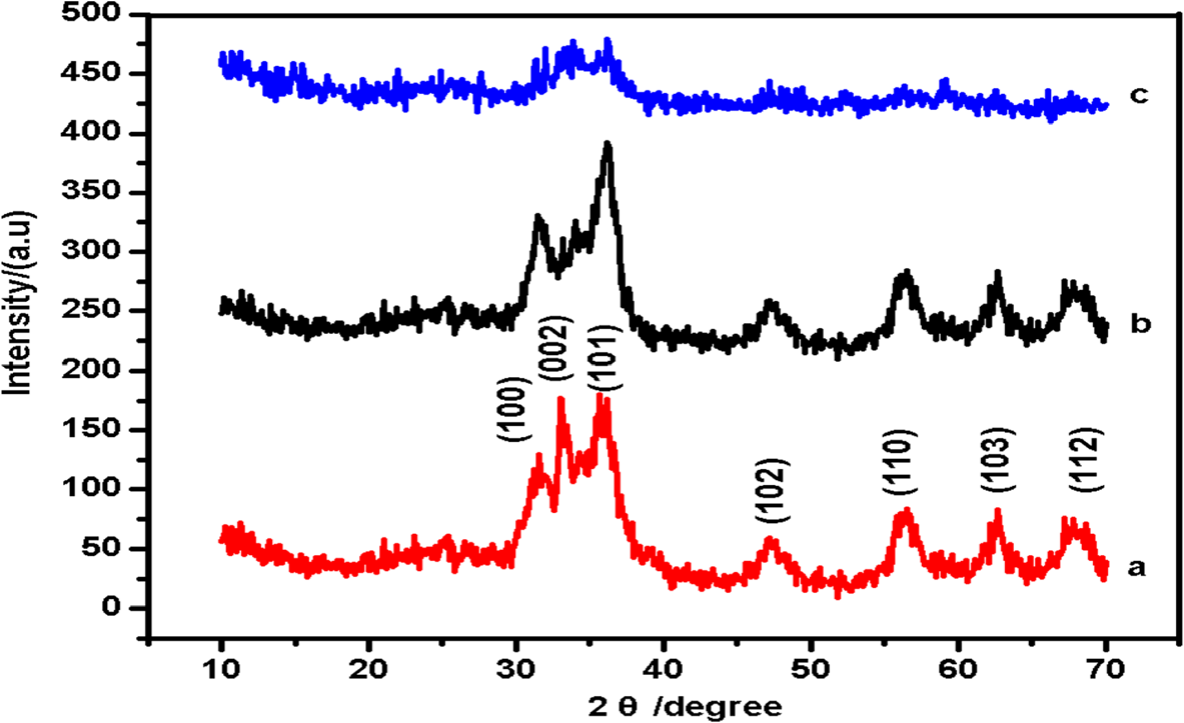
XRD patterns for (a) ZnO, (b) 5% Cd-doped ZnO and (c) TOPO/ODA-modified Cd-doped ZnO QDs.

TEM images of undoped ZnO QDs and ZnO QDs doped with 2%, 5%, 10% and 20% Cd are shown in Figure 1A,B,C,D,E, respectively. The images reveal that the particle sizes of the Cd-doped ZnO QDs are significantly less than those of undoped ZnO QDs. Furthermore, all of the QDs are circular in shape with diameters of approximately 3 to 6 nm, and the particle size is inversely proportional to the Cd concentration. These results were consistent with the XRD studies in demonstrating that doping with Cd suppresses the growth of ZnO QD particles and that the QDs possess a hexagonal wurtzite structure with space group *P6*_*3mc*_, as shown in Figure 2.

**Figure 3:**
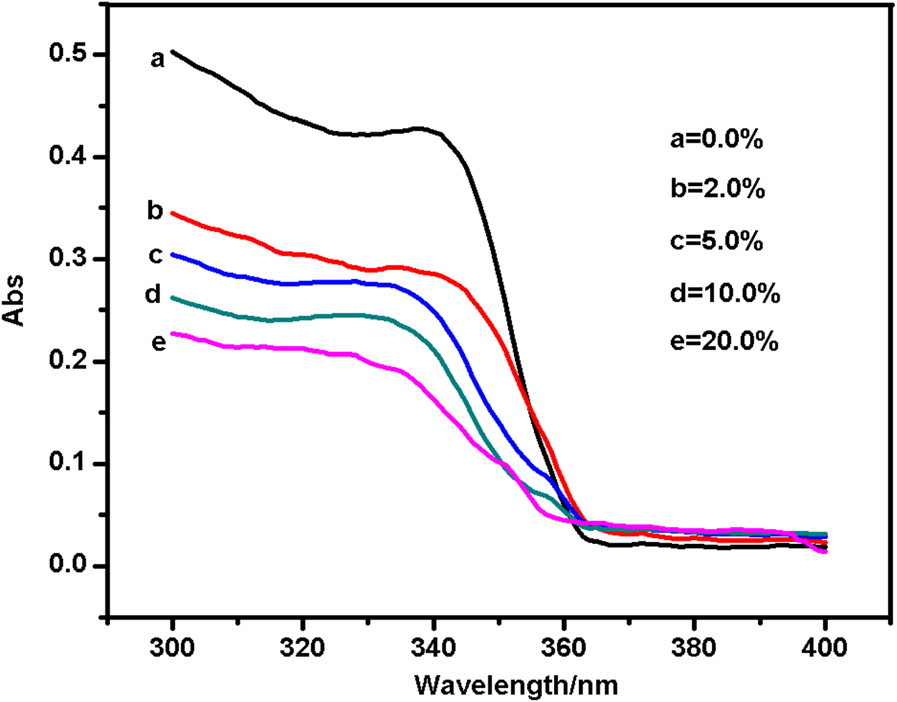
**The UV-visible absorption spectra of ZnO QDs with different concentrations of Cd.** (**a**) 0.0%, (**b**)2.0%, (**c**) 5.0%, (**d**) 10.0 (**e**) 20.0%.

**Figure 4:**
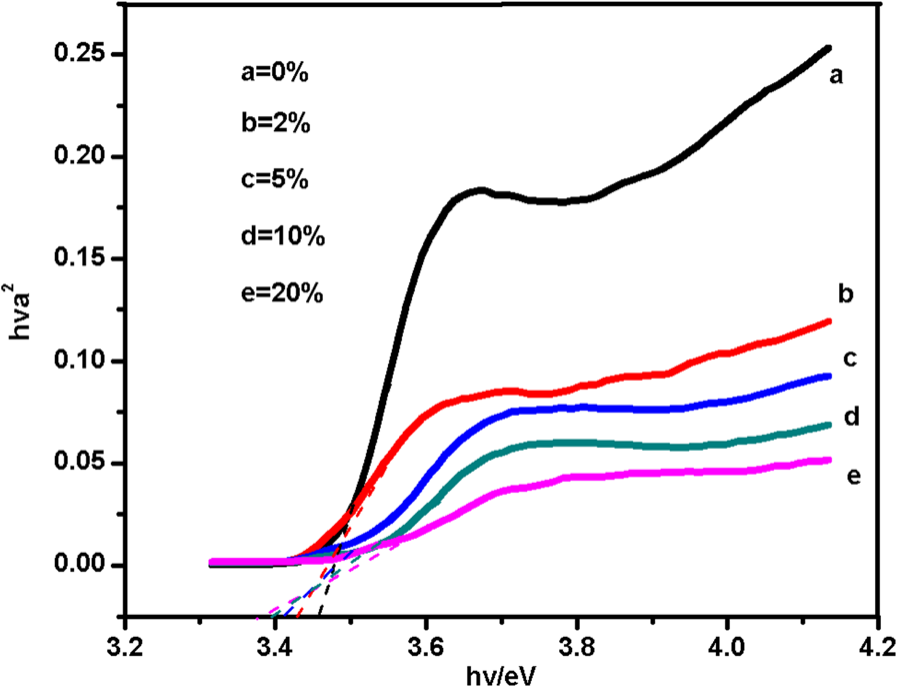
**Plots of (*α*hν)**^**2**^**versus hν of ZnO QDs with different concentrations of Cd.** (**a**) 0%, (**b**) 2%, (**c**) 5%, (**d**) 10%, (**e**) 20%.

Figure 3 shows the UV-visible absorption spectra of ZnO QDs with different concentrations of Cd. The ob-vious exciton absorption peak of ZnO QDs appears at approximately 340 nm due to the relatively large exciton binding energy of 60 meV at room temperature. For Cd-doped ZnO QDs, a blue shift was observed in the exciton absorption peak as the concentration of Cd was increased. This shift may be due to the quantum confinement effect. For the direct-band-gap semiconductor of ZnO, the band gap energy can be expressed by the following equation [3]:

(1) $${\left(\alpha \text{hν}\right)}^{2}=A(\text{hν}-\text{Eg}),$$

where *α* is the absorption coefficient, hν is the photon energy, *A* is the edge-width parameter and Eg is the band-gap energy for direct transitions as indicated in Figure 4. The particle size of the ZnO QDs with different concentrations of Cd can also be estimated from the band-gap energy based on the Brus effective mass approximation formula [14]. The diameters of these samples were calculated to be 6, 5.2, 3.6, 3.3 and 3.2 nm for pure ZnO, 2.0% Cd-doped ZnO, 5.0% Cd-doped ZnO, 10.0% Cd-doped ZnO and 20.0% Cd-doped ZnO QDs, respectively. The calculated diameters were consistent with those observed from the TEM analysis.

**Figure 5:**
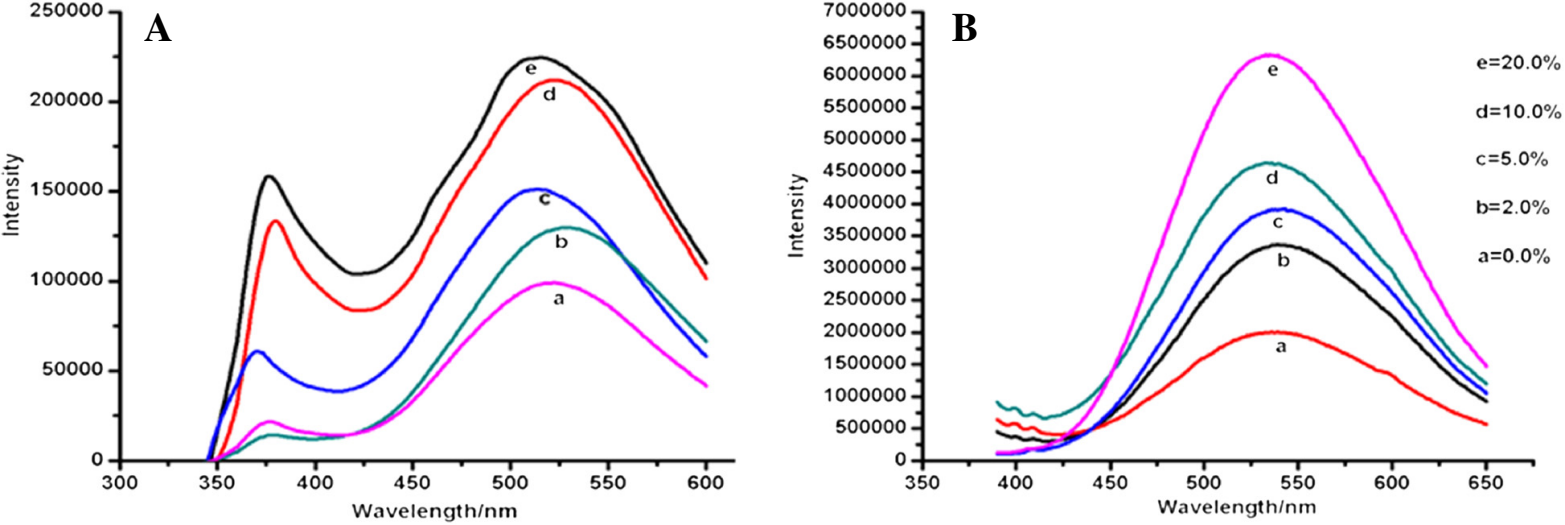
**Emission spectra of ZnO QDs with different concentrations of Cd.** (**A**) 318-nm and (**B**) 365-nm excitation. (a) 0.0%, (b) 2.0%, (c) 5.0%, (d) 10.0%, (e) 20.0%.

Figure 5A illustrates that the fluorescence spectra of ZnO QDs consist of two parts: one narrow and weak peak at 385 nm is present around the ultraviolet region due to the free exciton composite, which is caused by the recombination luminescence of electron–hole pairs from the hole at the top of the valence band and the electronic states at the bottom of the conduction band [15]; the other part is a strong and broad green emission peak at 525 nm in the visible region that arises due to the band gap of the intrinsic defect energy levels. The broad peak in the visible region is associated with structural defects, such as interstitials, oxygen vacancies and surface traps [16,17]. Previous studies have shown that the properties of UV fluorescence emission peaks depend strongly on the interband transitions and the exciton recombination. Emission intensity in the ultraviolet region is increased due to the incorporation of Cd, which changed the ZnO bandgaps and promoted recombination luminescence of the exciton. The emission spectra of the ZnO QDs with different concentrations of Cd at an excitation wavelength of 365 nm are shown in Figure 5B. The intensity of the visible light emission peak was significantly enhanced with increasing Cd concentration. The luminescence efficiency of nanoparticles is generally believed to strongly depend on the nature of the surface because large surface-to-volume ratios cause surface defects in smaller particles [18,19]. Because both Cd and Zn belong to the IIB family, they are similar in physical and chemical properties and have the same valence electron configuration: (*n –* 1)*d*^10^*ns*^2^. Furthermore, *R*_Cd2+_ (0.097 nm) is larger than *R*_Zn2+_ (0.074 nm). As a result, Cd ions displace some Zn ions in the ZnO lattice as substitutional impurities. Moreover, the incorporation of Cd changes the coordination number of the cations and permits more oxygen vacancies and other defects, which may be the main reason for the enhancement of the luminescence intensity with increased Cd doping.

**Figure 6:**
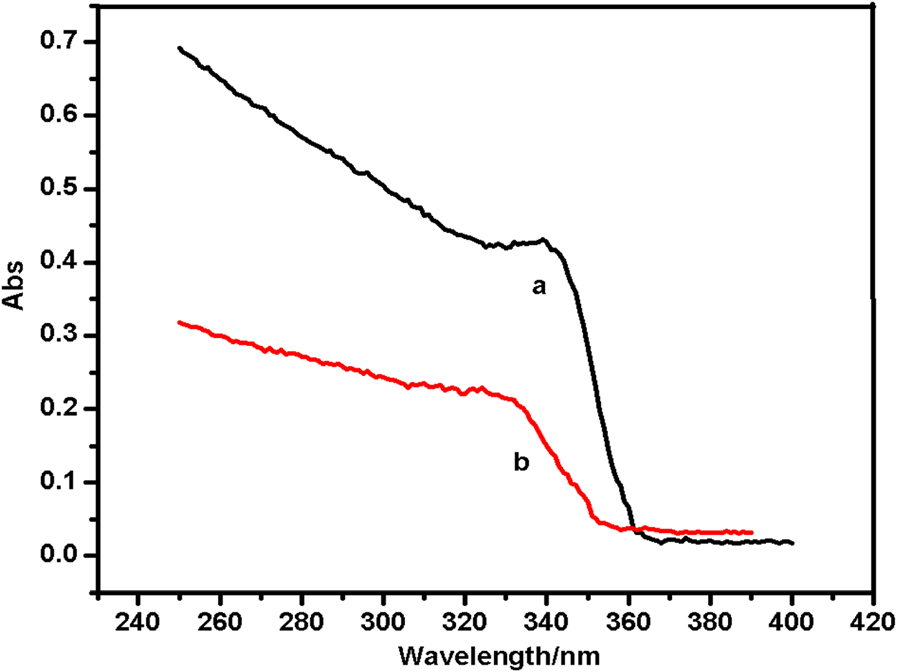
The UV-visible absorption spectra of (a) unmodified and (b) TOPO/ODA-modified Cd-doped ZnO QDs.

Figure 6 shows the UV-visible absorption spectra for both TOPO/ODA-modified and unmodified Cd-doped ZnO QDs. The exciton absorption peaks of both samples appear at approximately 340 nm, whereas a blue shift relative to the peak position of unmodified Cd-doped ZnO QDs was observed in the exciton absorption peak for the TOPO/ODA-modified Cd-doped ZnO QDs when the TOPO/ODA mass ratio was 1:2. In the unmodified system, the QD particles easily aggregated to form clusters, and the particle growth within these clusters caused the shift. Thus, we deduced that the introduction of TOPO/ODA into the QD preparation process can effectively inhibit the aggregation of particles.

**Figure 7:**
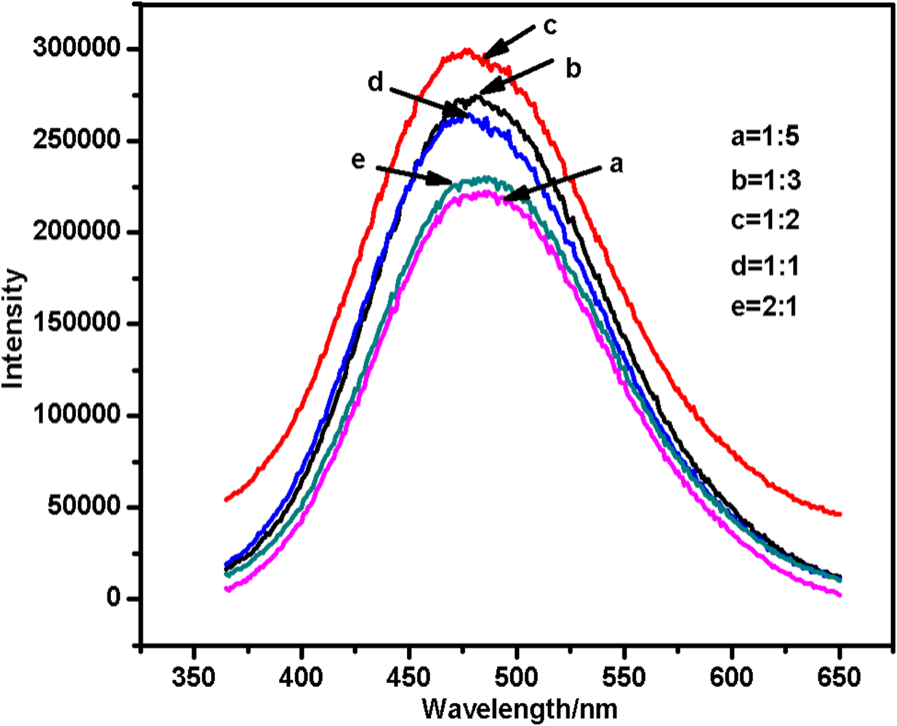
**Fluorescence spectra of Cd-doped ZnO QDs for different TOPO/ODA mass ratios.** (**a**)1:5, (**b**) 1:3, (**c**) 1:2, (**d**) 1:1, (**e**) 2:1.

We sought to obtain a full understanding of the effect of these modifications on the fluorescence properties of QDs. The emission spectra, which were derived from the excitation of Cd-doped ZnO QDs with different TOPO/ODA mass ratios at 341 nm, are shown in Figure 7. A shift similar to that of the UV absorption peaks was observed in the emission spectra because of the quantum confinement effect and because of the increase in the band-gap width. The results indicate that the coating of the surfaces of the QDs with TOPO and Cd, combined with the coordination effects of TOPO and cadmium ions, can enhance the luminous intensity. The luminous intensity was observed to increase as the TOPO/ODA mass ratio was increased when the ratio was 1:2 or less. Excessive TOPO adhesion to the QD particles can block the absorption of the excitation light [20], and the emission intensity decreases as a result. The results show that a 1:2 TOPO/ODA mass ratio is optimal for the generation of maximum luminous intensity.

**Figure 8:**
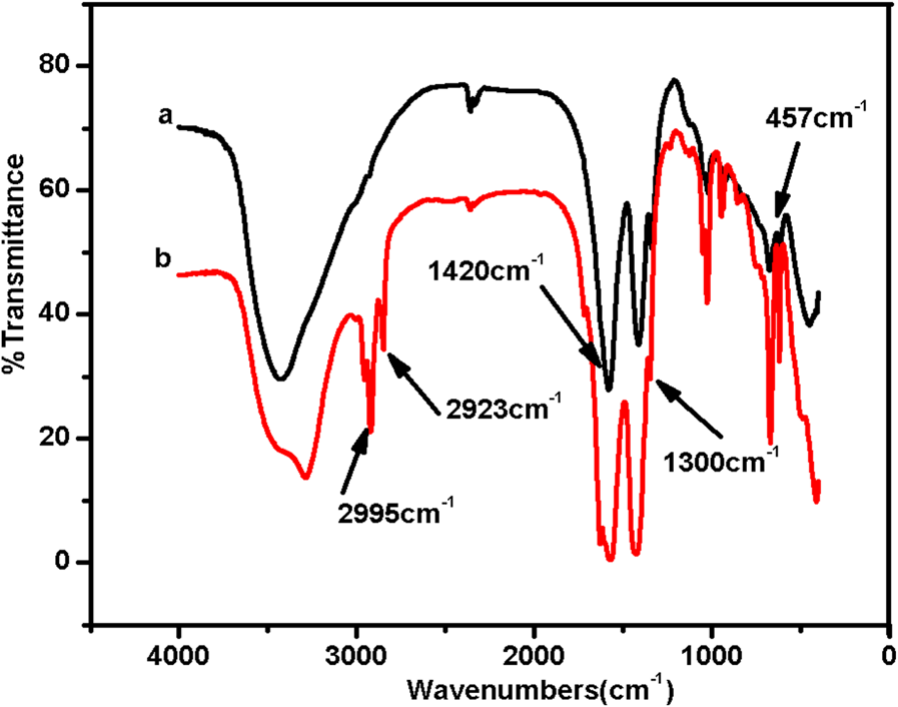
**FT-IR spectra of (a) unmodified and (b) TOPO/ODA**-**modified Cd-doped ZnO QDs.**

Figure 8 shows the FT-IR spectra of both the unmodified Cd-doped ZnO QDs and the TOPO/ODA-modified QDs. The well-known stretching mode of Cd = O was observed at 1,420 cm^−1^. Clear Zn-O-Zn stretching modes were observed at 457 cm^−1^[21], and these stretching modes were indicative of the successful synthesis of ZnO in both cases, as previously confirmed by XRD. A peak at 1,300 cm^−1^ was observed because of the absorption of P = O, whereas stretching modes at 2,995 and 2,923 cm^−1^ were due to the -CH_3_ and -CH_2_- groups present at the surface of the Cd-doped ZnO QDs embedded in TOPO/ODA. These results demonstrate that the polymer successfully coated the surface of the Cd-doped ZnO QDs.

Figure 2 presents representative XRD patterns of the ZnO QDs (a), the 5% Cd-doped ZnO QDs (b) and the TOPO/ODA-modified Cd-doped ZnO QDs (c) synthesised via the sol–gel method. The XRD spectra in Figure 2a,b revealed broad peaks at 31.63°, 34.50°, 36.25°, 47.50°, 56.60°, 62.80° and 67.92°; these diffraction peaks matched the JCPDS file for ZnO (JCPDS 36–1451) and were indexed as a hexagonal wurtzite structure of ZnO with space group *P6*_*3mc*_. Because no impurity peak associated with Cd clusters or CdO was detected, the wurtzite structure was not disturbed by the addition of small amounts of Cd^2+^ during the sample preparation. Therefore, the broadening of the XRD peaks (i.e., Scherrer broadening) gave a clear indication of the formation of nanosized ZnO. The particle diameters of the ZnO and the 5% Cd-doped ZnO QDs were estimated using the Debye-Scherer equation:

(2) $$D=\frac{0.9\lambda }{B\cos\theta_{B}},$$

where *D* is the particle size, *λ* is the wavelength of ra-diation used, *θ*_B_ is the Bragg diffraction angle and *B* is the peak width at half maximum [22]. The XRD data, along with the TEM data presented previously, establish that doping with Cd suppresses the growth of ZnO QD particles. Furthermore, the XRD peaks the diffraction profiles of TOPO/ODA-modified Cd-doped ZnO QDs were not as sharp as in the case of the 5% Cd-doped ZnO sample, which indicated that the polymer coated the surfaces of the Cd-doped ZnO QDs. These results were also consistent with those from the FT-IR analysis.

**Figure 9:**
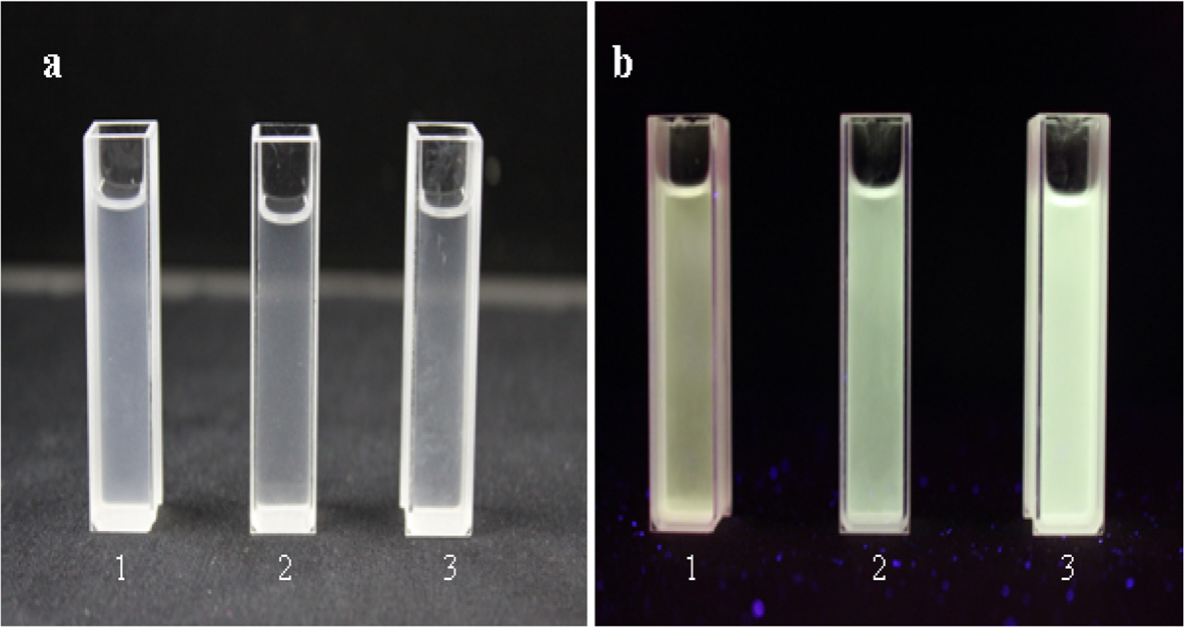
**Fluorescence photographs of QDs under illumination by (a) an ordinary lamp and (b) UV light.** (1) The ZnO QDs, (2) the 5% Cd-doped ZnO QDs, (3) the TOPO/ODA-modified Cd-doped ZnO QDs.

Figure 9a,b shows images of (1) the ZnO QDs, (2) the 5% Cd-doped ZnO QDs and (3) the TOPO/ODA-modified Cd-doped ZnO QDs illuminated with ordinary and UV (365-nm excitation) lamps, respectively. The samples under ordinary light exhibited a pure white colour, whereas the samples under UV light showed greenish-yellow luminescence. The fluorescence properties of the synthesised ZnO, the Cd-doped ZnO and the TOPO/ODA-modified Cd-doped ZnO QDs are shown in Figure 9. The luminous colour is apparently due to the emission peak at approximately 500 nm in the visible region, and this broad peak is associated with structural defects, such as interstitials, oxygen vacancies and surface traps.

## Conclusions

In conclusion, we have successfully synthesised QDs of ZnO, Cd-doped ZnO and TOPO/ODA-modified Cd-doped ZnO. Blue shifts of the UV absorption peaks of the Cd-doped ZnO QDs were observed with increasing Cd concentration, which indicated a reduction of the QD size and a strengthening of the quantum confinement effect after doping. Through various measurements, including UV, PL, XRD and FT-IR, the surfaces of the QDs were found to play an important role in their optical properties. Both the TOPO and Cd coatings on the ZnO QD surfaces and an excessive amount of TOPO led to a reduction in luminous intensity. Thus, the TOPO/ODA mass ratio strongly influences the luminous intensity. A mass ratio of 1:2 was found to be the optimal ratio for maximal luminous intensity. These results provide strong support for the further development of extensive optical device applications, including bio-imaging and bio-sensors, based on the conjugation of these nanocrystals to biological entities.

## Competing interests

The authors declare that they have no competing interests.

## Authors’ contributions

JZ performed the XRD, UV–vis and FT-IR experimental work and drafted the manuscript. SQZ was the thesis director. KZ was the project coordinator and analysed the fluorescence spectra. JQZ performed the TEM analysis. YFC participated in the design and coordination of the study. All authors read and approved the final manuscript.

## Authors’ information

Dr. SQZ is working as a professor in Guangdong University of Technology and is the director of the Department of Pharmacy Engineering. Professor Zhao has been studying about building immune determination method for toxic chemical with luminescent markers.

## References

[B1] Armon R Dosoretz C Starosvetsky J Orshansky F Saadi I Sol–gel applications in environmental biotechnology J Biotechnol 1996 51 279 285 10.1016/S0168-1656(96)01607-0 8988652

[B2] Yang H Luan W Tu S Wang ZM Synthesis of nanocrystals via microreaction with temperature gradient: towards separation of nucleation and growth Lab Chip 2008 8 451 455 10.1039/b715540a 18305864

[B3] Ghosh M Raychaudhuri AK Structure and optical properties of Cd-substituted ZnO (Zn1-xCdxO) nanostructures synthesized by the high-pressure solution route Nanotechnology 2007 18 115618 10.1088/0957-4484/18/11/115618

[B4] Sulieman KM Huang XT Liu JP Tang M Controllable synthesis and characterization of hollow-opened ZnO/Zn and solid Zn/ZnO single crystal microspheres Nanotechnology 2006 17 4950 4955 10.1088/0957-4484/17/19/029

[B5] Sun D Sue H-J Miyatake N Optical properties of ZnO quantum dots in epoxy with controlled dispersion J Phys Chem C 2008 112 16002 16010 10.1021/jp805104h

[B6] Vijayalakshmi S Venkataraj S Jayavel R Characterization of cadmium doped zinc oxide (Cd: ZnO) thin films prepared by spray pyrolysis method J Phys D Appl Phy 2008 41 245403 10.1088/0022-3727/41/24/245403

[B7] Wang F Liu B Zha Z Yuan S Synthesis and properties of Cd-doped ZnO nanotubes Physica E Low Dimens Syst Nanostruct 2009 41 879 882 10.1016/j.physe.2008.12.026

[B8] Passmore BS Wu J Manasreh M Kunets VP Lytvyn P Salamo G Room temperature near-infrared photoresponse based on interband transitions in In0.35Ga0.65As multiple quantum dot photodetector IEEE Electron Device Lett 2008 29 224 227

[B9] Wu J Makableh Y Vasan R Manasreh M Liang B Reyner C Huffaker D Strong interband transitions in InAs quantum dots solar cell Appl Phys Lett 2012 100 051907 10.1063/1.3681360

[B10] Wenas WW Yamada A Takahashi K Yoshino M Konagai M Electrical and optical properties of boron-doped ZnO thin films for solar cells grown by metalorganic chemical vapor deposition J Appl Phys 1991 70 7119 10.1063/1.349794

[B11] Subramanyam TK Rao GM Uthanna S Process parameter dependent property studies on CdO films prepared by DC reactive magnetron sputtering Mat Chem Phys 2001 69 133 142 10.1016/S0254-0584(00)00376-X

[B12] Choi YS Lee CG Cho SM Transparent conducting ZnxCd1 − xO thin films prepared by the sol–gel process Thin Solid Films 1996 289 153 158 10.1016/S0040-6090(96)08923-7

[B13] Zou L Ye ZZ Huang JY Zhao BH Structural characterization and photoluminescent properties of Zn1-xMgxO films on silicon Chin Phys Lett 2002 19 1350 1352 10.1088/0256-307X/19/9/341

[B14] Guo L Yang SH Yang CL Yu P Wang JN Ge WK Wong GKL Highly monodisperse polymer-capped ZnO nanoparticles: preparation and optical properties Appl Phys Lett 2000 76 2901 2903 10.1063/1.126511

[B15] Mei Z Zhu YH Lee WB Yue TM Pang GKH Microstructure investigation of a SiC whisker reinforced eutectoid zinc alloy matrix composite Composites Part A 2006 37 1345 1350 10.1016/j.compositesa.2005.08.011

[B16] Huang MH Wu YY Feick H Tran N Weber E Yang PD Catalytic growth of zinc oxide nanowires by vapor transport Adv Mat 2001 13 113 116 10.1002/1521-4095(200101)13:2<113::AID-ADMA113>3.0.CO;2-H

[B17] Liu M Kitai AH Mascher P Point defects and luminescence centres in zinc oxide and zinc oxide doped with manganese J Lumin 1992 54 35 42 10.1016/0022-2313(92)90047-D

[B18] Rani S Suri P Shishodia PK Mehra RM Synthesis of nanocrystalline ZnO powder via sol–gel route for dye-sensitized solar cells Sol Energy Mater Sol Cells 2008 92 1639 1645 10.1016/j.solmat.2008.07.015

[B19] Wu XL Siu GG Fu CL Ong HC Photoluminescence and cathodoluminescence studies of stoichiometric and oxygen-deficient ZnO films Appl Phys Lett 2001 78 2285 2287 10.1063/1.1361288

[B20] Zhuang J Liu M Liu H MAA-modified and luminescence properties of ZnO quantum dots Sci in China Ser B 2009 52 2125 2133 10.1007/s11426-009-0198-5

[B21] Zhao B Zhao S Zhang K Liu D Su J Preparation and characterization of thiourea modified water-soluble Cd doped ZnO quantum dots Acta Chim Sinica 2011 69 777 782

[B22] Wang YS Thomas PJ O’Brien P Optical properties of ZnO nanocrystals doped with Cd, Mg, Mn, and Fe ions J Phys Chem B 2006 110 21412 21415 10.1021/jp0654415 17064087

